# Sequencing and comparative analyses of ‘Candidatus Phytoplasma solani’ genomes reveal diversity of effectors and potential mobile units

**DOI:** 10.1099/mgen.0.001401

**Published:** 2025-04-28

**Authors:** Martina Šeruga Musić, Bruno Polak, Marina Drčelić, Shen-Chian Pei, Chih-Horng Kuo

**Affiliations:** 1 Department of Biology, Faculty of Science, University of Zagreb, Zagreb, Croatia; 2 Institute of Plant and Microbial Biology, Academia Sinica, Taipei, Taiwan, ROC

**Keywords:** effector proteins, mobile genetic elements, pathogenicity, phytoplasma, plant pathogens, virulence factors

## Abstract

Phytoplasmas (genus ‘*Candidatus* Phytoplasma’) encompass a group of uncultivated bacteria affecting numerous plant species and causing significant damage in agriculture worldwide. They have a dual parasitic cycle, including colonization of both plant phloem and insect cells. Their genomes are small, diverse, repetitive, prone to rearrangements and harbour transposon-like elements known as potential mobile units (PMUs). In the Euro-Mediterranean region, ‘*Ca*. P. solani’ is an important species due to its broad range of plant hosts and insect vectors. To provide insights into the genomic diversity of this species, particularly the repertoire of putative effectors and PMUs, this study conducted genome sequencing and analyses of two *‘Ca*. P. solani’ strains originating from different plants and transmitted by different insects. Based on *de novo* assembly, we obtained 19 contigs totalling 656 141 bp for strain STOL and 28 contigs totalling 707 036 bp for strain ST19. The prevalence of repetitive sequences and PMUs contributed to the fragmentation of these draft assemblies. The annotation identified 28 and 26 genes that encode putative secreted proteins in these two strains, respectively, including several homologues of previously characterized phytoplasma effectors. Our comparative analyses further identified species- and strain-specific genes. Frequently, genes that encode putative secreted proteins and effectors were found within PMU-like regions in both genomes. Moreover, strain STOL showed characteristics of a more reduced genome, having fewer PMU-like repetitive elements and genome rearrangements, while strain ST19 exhibited a higher level of sequence divergence in its PMU genes. The high levels of genomic diversity among ‘*Ca*. P. solani’ strains suggested rapid evolution of this species, which may contribute to its wide host range and adaptability potential. This study provides novel data on the diversification of ‘*Ca*. P. solani’ genomes. These results provide a foundation for future functional studies of putative effectors and their interactions with host targets, which could facilitate deciphering the pathogenicity strategies of this successful and versatile pathogen.

**Impact Statement**

Wall-less bacteria known as phytoplasmas (genus ‘*Candidatus* Phytoplasma’) encompass a diverse group of endocellular pathogens affecting numerous plant species, including important crops worldwide. While the economic importance is increasing, studies related to phytoplasma genomics are still limited due to the inability to cultivate them axenically. In the Euro-Mediterranean region, ‘*Ca*. P. solani’ is an important pathogen with a wide range of plant hosts and insect vectors. Within this species, strains have variable genome sizes, but detailed understanding is lacking. In this study, we present results on genome sequencing of two new strains, followed by comparative analyses of effector genes and putative mobile genetic elements. The findings demonstrate high levels of genomic diversity, suggesting that this species has a rapid evolutionary rate, which may contribute to its wide host range and adaptability potential. In addition to the novel knowledge, this work could facilitate future functional studies of pathogenicity mechanisms, thereby accelerating the development of control strategies against this versatile pathogen.

## Data Summary

The datasets generated from this study are deposited in the National Center for Biotechnology Information (NCBI) databases under the following accession numbers. ‘*Ca*. P. solani’ strain ST19: BioProject PRJNA1135947 (https://www.ncbi.nlm.nih.gov/bioproject/PRJNA1135947); raw reads SRR29858978 (https://www.ncbi.nlm.nih.gov/sra/SRR29858978) and SRR29858979 (https://www.ncbi.nlm.nih.gov/sra/SRR29858979); assembly JBFSHS000000000.1 (https://www.ncbi.nlm.nih.gov/nuccore/JBFSHS000000000.1/). ‘*Ca*. P. solani’ strain STOL: Bioproject PRJNA1135948 (https://www.ncbi.nlm.nih.gov/bioproject/PRJNA1135948); raw reads SRR29858980 (https://www.ncbi.nlm.nih.gov/sra/SRR29858980) and SRR29858981 (https://www.ncbi.nlm.nih.gov/sra/SRR29858981); assembly JBFPNQ000000000.1 (https://www.ncbi.nlm.nih.gov/nuccore/JBFPNQ000000000.1/).

The authors confirm all supporting data, code and protocols have been provided within the article or through supplementary data files.

## Introduction

Phytoplasmas (genus ‘*Candidatus* Phytoplasma’) are important endocellular pathogens that affect many plant species worldwide and cause substantial losses in agriculture [1,3]. This diverse group of uncultivable wall-less bacteria have a specific dual parasitic cycle, which involves the colonization of plant phloem and phloem-feeding insects. In plants, phytoplasmas cause profound disturbances of developmental and metabolic processes. The disturbances result in severe symptoms such as abnormal flower formation, yellowing or reddening of leaves, hypertrophic growth of axillary shoots (known as witches’-broom symptom), stunting and die-back. Despite the challenges in cultivation of phytoplasmas in axenic culture, about 20 complete genome assemblies and more than 200 draft genome assemblies are available for these bacteria [4] (https://www.ncbi.nlm.nih.gov/datasets/genome/?taxon=33926). The first complete assemblies of the phytoplasma genome revealed that their genomes are small and reduced, but often contain substantial amounts of repetitive sequences and multicopy genes that in some cases could disable the full assembly of genome drafts into a single contig [5,7]. Regarding the genome size, as other members of the class Mollicutes such as mycoplasmas, acholeplasmas and spiroplasmas, phytoplasmas have a considerable variation within the genus, with genome range from 530 to 1350 kbp, where some species such as ‘*Ca*. P. asteris’ and ‘*Ca*. P. solani’ show particular heterogeneity among strains [4,8]. This heterogeneity could be attributed to the presence of repetitive sequences organized in specific genetic elements termed potential mobile units (PMUs) [6]. These transposon-like replicative regions of up to 20 kb, prone to horizontal gene transfer and genetic rearrangements, are suggested to have a role in the extreme adaptability of phytoplasma strains to different plant and insect hosts. They are characterized by carrying genes encoding proteins involved in DNA transposition and replication such as *ssb*, *tmk*, *tra5*, *dnaB* and *dnaG*, but often encompass genes for putative secreted proteins, effectors or other potential virulence factors [6,7, 9]. Additionally, these mobile genetic elements were found to be involved in horizontal gene transfer [10] and strongly associated with the genome size variation at both within- and between-species levels [11].

In phytopathogenic bacteria, effectors are most commonly small proteins that affect plant physiology, signalling pathways and thus modify the interactions of plants with insect vectors as well. As phytoplasmas are endocellular pathogens, unlike in most phytopathogenic Gram-negative bacteria, their effectors are secreted by Sec-dependent transport systems directly into the host cell cytoplasm. Until now, only a small number of phytoplasma effectors have been studied in detail, and their mechanisms and interaction with plant hosts have been deciphered. In early works, these effectors were initially studied based on ‘*Ca*. P. asteris’ strains AY-WB and OY. Notable examples include secreted AY-WB proteins (SAP) SAP11 and SAP54, TENGU and PHYL1 [12,17]. More recently, mechanisms of several SAP homologues and other effectors from other phytoplasma species have also been characterized [18,22]. It was found that phytoplasma effectors deeply disturb plant developmental processes and defence mechanisms, leading to the appearance of characteristic symptoms [12,22]. In the molecular phytoplasma–plant interactions, diverse plant transcription factors that regulate immune responses and development have been identified as the main targets of phytoplasma effectors. Moreover, the investigation of host–pathogen protein interaction networks provided further insights into the unique and specific pathogenic strategies utilized by phytoplasmas [23].

In this work, we focus on ‘*Ca*. P. solani’, an economically important phytoplasma infecting many crops such as grapevine, potato, tomato, maize and sugar beet. This pathogen is considered endemic to the Euro-Mediterranean area but sporadically detected worldwide. It encompasses numerous strains, with some being transmitted to a broad range of plants by different polyphagous insects, while some have a limited host range and insect vector transmissibility [24,25]. The variation was assumed to be linked to the genome plasticity. In our previous work, we identified 38 putative effectors in the genome of ‘*Ca*. P. solani’ strain SA-1 [7]. To improve our understanding of the genomic variation among strains, we conducted whole-genome sequencing and analysis of two additional ‘*Ca*. P. solani’ strains. These two strains originated from different plant hosts and were transmitted by different insect vectors. Importantly, strain STOL included in this work is the reference strain of ‘*Ca*. P. solani’ [3]. Therefore, the newly generated genomic information from this study provides a valuable foundation for future comparative genomics among phytoplasmas.

## Methods

### Biological materials and DNA preparation

Isolation of total genomic DNA was performed by OmniPrep for Plant commercial kit (G-Biosciences, St. Louis, MO, USA) and modified CTAB procedure as described previously [26] from 0.1 g and 0.25 g of Madagascar periwinkle (*Catharanthus roseus* L. Don.) tissue, respectively. Periwinkle plants were infected by one single ‘*Ca*. P. solani’ strain (ST19 or STOL) originating from different plant hosts and maintained by grafting. Both strains were transferred to periwinkle by insect vectors from the original host. Strain ST19 was transferred by *Hyalestes obsoletus* collected from the original natural host *Urtica dioica* L. (nettle) in July 2020 at location Sveti Ivan Žabno, Croatia (N45 57.516 E16 39.219), while the strain STOL was transferred by *Reptalus panzeri* (male specimens) from the original natural host *Zea mays* L. (maize) in July 2016 at location Dobanovci, Serbia (N44 48.996 E20 15.127). The concentration and purity of DNA after extraction were determined by using Nanodrop 2000c spectrophotometer (Thermo Fisher Scientific, USA) and Qubit 1.0 fluorometer (Thermo Fisher Scientific, USA).

### Library preparation and sequencing

For both strains, one Illumina and one Oxford Nanopore Technologies (ONT) library was prepared for sequencing. For Illumina, shotgun paired-end libraries were constructed and sequenced by using the Illumina MiSeq 2×250 bp configuration (Illumina) by GENEWIZ Azenta Life Sciences (Burlington, MA 01803, USA). For ONT, the library preparation and MinION sequencing (FLO-MIN106D SpotON Flow Cell Mk I R9 Version) were performed by a core facility in Academia Sinica (Taipei, Taiwan) according to the procedures described in our previous study [11].

### Assembly and annotation

The procedure for assembly and annotation was modified from those described in our previous studies of phytoplasma genomes [10,11]. All bioinformatics tools were used with the default settings unless stated otherwise. Briefly, the Illumina reads were mapped to nuclear (GenBank accession: JQHZ00000000.1) and chloroplast genome (KC561139) of the plant host using BWA v. 0.7.17 [27] for removing contaminations. The ONT reads were mapped to the genome of ‘*Ca*. P. solani’ strain SA-1 (MPBG00000000.1) using Minimap2 [28] to identify phytoplasma-derived reads. The filtered reads were used for hybrid *de novo* assembly using Unicycler v. 0.4.9b [29]. After the initial assembly, all contigs were used to run BLASTN and BLASTX searches against NCBI non-redundant nucleotide and protein databases to identify putative phytoplasma contigs. For validation, all raw reads were mapped to the filtered contigs to identify and correct assembly artefacts.

For annotation, the prediction of rRNA, tRNA and protein-coding genes was conducted by using RNAmmer v. 1.2 [30], tRNAscan-SE v. 1.3.1 [31] and Prodigal v. 2.6.3 [32], respectively. The predicted coding sequences (CDSs) were further checked with OrthoMCL [33], BlastKOALA and BLAST against NCBI nr db, followed by manual curation with the result of NCBI Microbial Genome Submission Check. Prediction of putative effector genes was performed by SignalP v. 5.0 [34] using the default settings, followed by processing of mature protein sequences without the signal peptide by using TMHMM v. 2.0 [35] to identify transmembrane domains. Signal peptide length between 21 and 52 amino acids was chosen to identify putative secreted proteins. Furthermore, all candidates without any transmembrane domain were manually examined to remove those with other well-annotated functions (e.g. ABC transporter).

### Genome comparisons

To assess the overall genome relatedness index (OGRI) among ‘*Ca*. P. solani’ strains, we utilized fastANI v. 1.33 [36] to quantify alignment fraction (AF; proportion of genomic regions shared) and average nucleotide identity (ANI). Whole genome alignment of the three ‘*Ca*. P. solani’ strains was performed by using the progressive Mauve algorithm [37].

OrthoVenn3 software [38], an effective and user-friendly online tool for comparative genomics research, was used for comparison of orthologous clusters among representative phytoplasma species and strains, as well as for visualization of results. Protein sequence files of the following representative phytoplasma genomes were retrieved from NCBI and used for the analyses: ‘*Ca*. P. solani’ strain SA-1 (MPBG01000000), ‘*Ca*. P. australiense’ strain PAa (AM422018), ‘*Ca*. P. asteris’ strain AY-WB (CP000061), *‘Ca*. P. asteris’ strain OY (AP006628), *‘Ca*. P. mali’ (CU469464) and *flavescence dorée* (FD) phytoplasma strain CH (CP097583) [37].

### Phylogenetic analyses of selected PMU-related and effector genes

Multiple alignments of nucleotide and amino-acid sequences were done by using the ClustalX programme [39]. Subsequent phylogenetic analyses were performed by maximum-likelihood method mega11 [40] for individual gene trees or OrthoVenn3 for the species tree.

For selected putative effector genes, including SAP11-like and SAP54-like (AB2N29_0110), specific primers were designed by using Geneious Prime 2024.0.7 software (https://www.geneious.com) to assess their presence and variability in ‘*Ca*. P. solani’ strains ST19, STOL and SA-1. The amplicons were sequenced (GENEWIZ Azenta Life Sciences), followed by editing of raw sequences by Geneious Prime 2024.0.7; alignment and phylogenetic analyses were performed as described above. The primer sequences and PCR conditions used are given in Table S1 (available in the online version of this article).

## Results

### Genome assembly and general features of ‘*Ca*. P. solani’ strains st19 and stol genomes

The ONT sequencing produced approximately 1.3 GB raw reads for ST19 and 0.7 GB for STOL, providing approximately 18- and 20-fold sequencing depth coverage, respectively. The Illumina sequencing produced approximately 1.6 GB raw reads for each strain, providing approximately 11- and 81-fold coverage for ST19 and STOL, respectively. The differences in coverage were caused by the differences in the proportion of phytoplasma reads in these samples. Combining the ONT and Illumina reads, the hybrid *de novo* assembly generated draft genomes with total size of 707 036 bp (ST19) and 656 141 bp (STOL) in 28 and 18 contigs, respectively. The contig length ranges from 1386 to 114 688 bp and a N50 value of 49 881 bp for ST19, while the STOL draft assembly has a N50 value of 83 092 bp with the contig length ranges from 1228 to 158 078 bp (Table 1). A substantial number of tRNA genes, as well as one or two rRNA operons, were found in these assemblies, indicating their high levels of completeness (Table 1). The number of annotated full-length CDSs is 639 for ST19 and 569 for STOL. However, strain STOL had only 187 CDSs annotated as hypothetical proteins as compared to 258 in ST19 strain, while the numbers of CDSs having an assigned function were similar (382 for STOL and 381 for ST19; Table 1). These findings provide quantitative estimates on the variability in size and gene content among ‘*Ca*. P. solani’ genomes.

**Table 1.** General features of ‘*Ca*. P. solani’ ST19 and STOL strains’ genomes and comparison to the selected representative phytoplasma genomes

Phytoplasma strain	'*Ca*. P. solani'			'*Ca*. P. asteris'	*Ca.* P. australiense’	‘*Ca.* P. mali’	***Flavescence dorée***
	STOL	ST19	SA-1	AY-WB	PAa	AT	CH
**16S rRNA group**	16SrXII			16SrI	16SrXII	16 SrX	16SrV
**No. of contigs**	18	28	19	1	1	1	1
**Chromosome size/combined length (bp**)	656.141	707.036	821.322	706.569	879.324	601.943	654.223
**Longest contig length (bp**)	158.078	114.688	138.876	n/a	n/a	n/a	n/a
**Shortest contig length (bp**)	1.228	1.386	1.801	n/a	n/a	n/a	n/a
**N50 (bp**)	83.092	49.881	76.256	n/a	n/a	n/a	n/a
**G+C content (%**)	28.2	28.3	28.3	26.9	27	21.4	21.7
**No. of CDSs**	569	639	709	671	839	497	506
**CDSs with assigned function**	382	381	452	450	502	338	350
**CDSs annotated as hypothetical proteins**	187	258	257	221	337	159	156
**No. of tRNA genes**	30	31	32	32	32	32	32
**No. of rRNA operons**	1	2	2	2	2	2	2
**GenBank accession number and reference paper**	JBFPNQ000000000 (this paper)	JBFSHS000000000 (this paper)	MPBG01000000 [7]	CP000061 [6]	AM422018 (Tran-Nguyen *et al*. 2008)	CU469464 [43]	CP097583 [44]

n/a – not applicable.

### Comparative genomic analysis and whole genome alignments

To confirm the species assignment and to assess the levels of genomic differentiation, strain STOL, the reference for ‘*Ca*. P. solani’ [3], was used for comparisons with other strains. The previously studied strain SA-1 shares 83.8% AF and 99.1% ANI. Another newly sequenced strain, ST19, shares 86.5% AF and 98.9% ANI. Based on the recently revised guidelines of using 95% ANI to classify ‘*Ca*. P.’ species, all of these strains can be confirmed as members of ‘*Ca*. P. solani’.

**Fig. 1.**
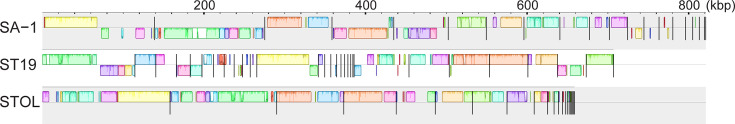
Whole genome alignment of three ‘*Ca*. P. solani’ strains. Strain STOL was used as the reference. The coloured blocks indicate syntenic regions among these genomes, those on the bottom represent inverted segments in the opposite orientation relative to the reference. Black vertical bars denote breakpoints between contigs. A scale bar is provided at the top of the alignment.

Whole genome alignment of these three ‘*Ca*. P. solani’ strains showed large blocks of syntenic regions (Fig. 1), despite all three assemblies being drafts, further supporting their close evolutionary relationships. However, although having only ~1% nucleotide sequence divergence, the presence of inverted regions and strain-specific regions were found. These regions encompass mostly genes corresponding to putative effectors, hypothetical proteins and PMU components such as *tra5*. Among these strains, STOL has the smallest genome size (Table 1) and the fewest strain-specific regions (Fig. 1).

**Fig. 2.**
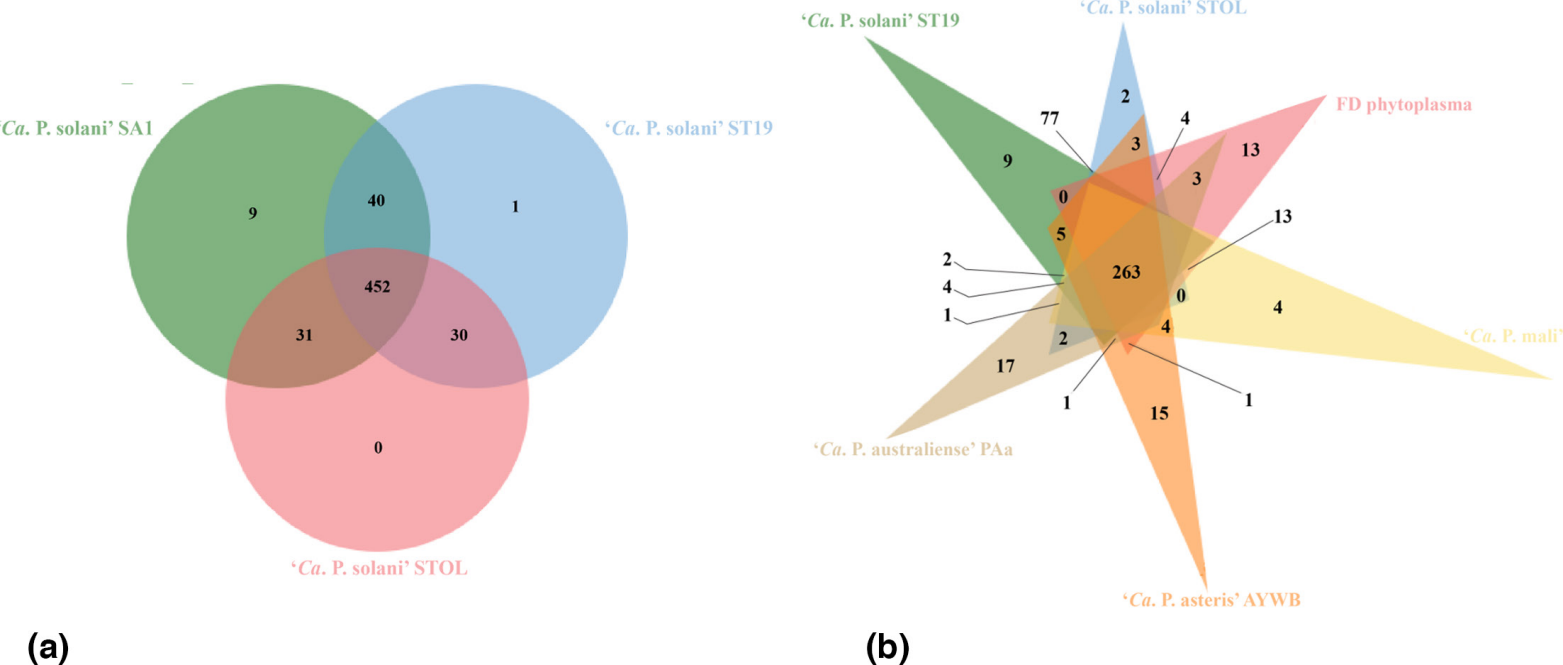
Venn diagrams showing the number of shared and unique orthologous gene clusters. (**a**) Among ‘*Ca*. P. solani’ strains SA-1, ST19 and STOL. (**b**) Among ‘*Ca*. P. solani’ strains ST19 and STOL, ‘*Ca*. P. australiense’ PAa, ‘*Ca*. P. asteris’ AY-WB, ‘*Ca*. P. mali’ and FD phytoplasma.

Comparative analyses of ‘*Ca*. P. solani’ strains ST19, STOL and SA-1 showed that they share a total of 452 orthologous gene clusters (Fig. 2a). Genes within these conserved clusters are mostly part of metabolic processes (73.41%; including RNA and heterocycle metabolic processes). ST19 has a single unique cluster with two proteins annotated as DNA (cytosine-5)-methyltransferase, which are involved in gene expression regulation, chromosomal DNA replication and repair control mechanisms. There are nine clusters found exclusively in SA-1 that contain all hypothetical proteins. In STOL, no unique cluster was found. Interestingly, among the 30 clusters shared by ST19 and STOL, these clusters mostly encompassed genes characteristic for PMU-related regions, such as *hflB*, *dnaB* and HIT family proteins.

**Fig. 3.**
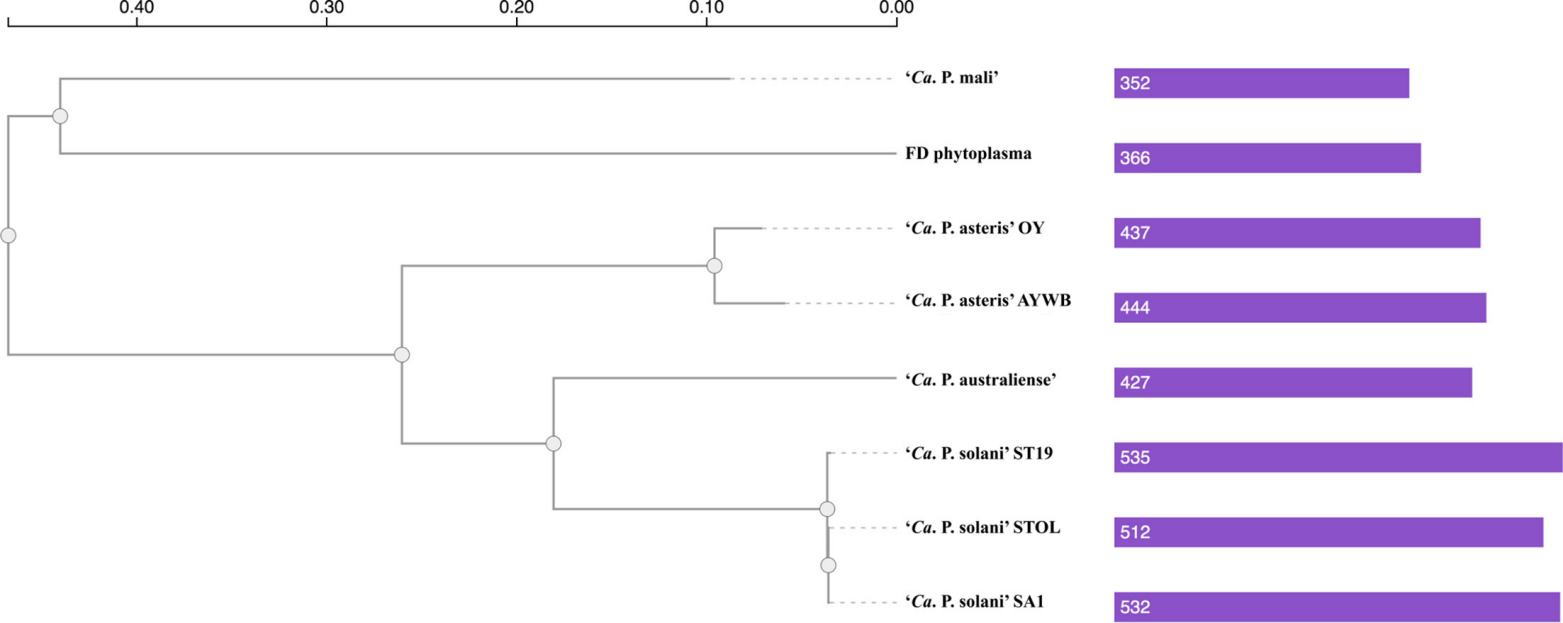
Phylogenetic tree showing the evolutionary relationships of ‘*Ca*. P. solani’ strains with the other representative phytoplasmas. The tree was based on the analysis of 255 single-copy orthologous proteins and constructed by maximum-likelihood method with FastTree2 within OrthoVenn3. The scale bar indicates the number of amino acid substitutions per site. The numbers and bar graph on the right indicate the number of orthologous clusters in each genome.

For comparisons with other more distantly related phytoplasmas, we included *‘Ca*. P. australiense’, ‘*Ca*. P. mali’, ‘*Ca*. P. asteris’ and FD phytoplasma to investigate their gene content (Table 1). A total of 263 orthologous gene clusters were shared by all strains (Fig. 2b). Phylogenetic analyses performed based on 255 conserved single-copy genes illustrated their evolutionary relationships (Fig. 3). Within this analysed group, there were two clusters found exclusively in ‘*Ca*. P. solani’ STOL and nine clusters found exclusively in ‘*Ca*. P. solani’ ST19. Moreover, ST19 shared five gene clusters with ‘*Ca*. P. asteris’ AY-WB, two clusters with ‘*Ca*. P. mali’, four with ‘*Ca*. P. australiense’, while none with FD phytoplasma. Similarly, STOL shared three gene clusters with ‘*Ca*. P. asteris’ AY-WB, two with ‘*Ca*. P. australiense’, four with FD phytoplasma and none with ‘*Ca*. P. mali’ (Fig. 2b). For genes involved in biological processes, the majority of shared genes (77.24%) among all analysed phytoplasmas are involved in various types of metabolism. For molecular function, nearly 30% of shared genes encode for proteins involved in nucleic acid binding [7].

### Effectors and PMU-like regions

Prediction of putative effector genes in ST19 and STOL identified 28 and 26 candidates, respectively. Among them, some are SAP homologues [6] such as SAP19-like, SAP39-like, SAP40-like, SAP44-like, SAP50-like, SAP53-like, SAP54-like, SAP55-like, SAP59-like, SAP61-like, SAP64-like and SAP65-like (Table S2). Subsequent comparative analyses showed that some of the predicted SAP-like genes were found in both genomes as well as in ‘*Ca*. P. solani’ strain SA-1 [41], including SAP39-like, SAP50-like, SAP53-like and SAP61-like. They shared very high amino acid sequence identity (96.77%, 99.72%, 94.49% and 94.87%, respectively) but distinct from those found in other phytoplasmas, hence were considered to be SAP homologues specific to ‘*Ca*. P. solani’. Several predicted effectors and hypothetical proteins had no homologues in other phytoplasmas and were present only in ‘*Ca*. P. solani’ strain SA-1 or ST19, and could be considered as strain-specific (Fig. 2b; Table S2 in Supplementary Material) [7].

Interestingly, homologues of SAP11 effector were found in all phytoplasmas with complete genome assemblies, as well as in the draft genome assembly of ‘*Ca*. P. solani’ SA-1, but not in the draft genome assemblies of ‘*Ca*. P. solani’ ST19 and STOL. We suspect that the absence in ST19 and STOL may be due to the localization of the gene within the genome region that is difficult to assemble. Therefore, we designed specific primers in order to assess the presence of SAP11-like in ST19 and STOL. The PCR amplification of the SAP11-like fragment was successful from all three ‘*Ca*. P. solani’ strains (Fig. S1A in Supplementary Material). Sequencing of these amplicons revealed 100% identical sequence in all of the analysed strains, which shares 87.94% nucleotide sequence identity to the homologue in ‘*Ca*. P. rubi’, 87.41% to ‘*Ca*. P. ziziphy’ and 81.93% to ‘*Ca*. P. asteris’ OY-M. Phylogenetic analyses of amino-acid sequences confirmed that the SAP-11-like effector of ‘*Ca*. P. solani’ is closely related to the homologue in ‘*Ca*. P. asteris’ OY-M (Fig. S2A in Supplementary Material).

Moreover, homologues of SAP54 effector were found as two variants, one in STOL (locus tag: AB2N29_0110; Table S2 in Supplementary Material) and the other in ST19 (AB2N28_4360; Table S2 in Supplementary Material). BLAST analyses showed that AB2N29_0110 shares 74.36% amino acid sequence identity to the homologue in phytoplasma ribosomal group 16SrXIII, 54.40% to ‘*Ca*. P. trifolii’, 56.80% to ‘*Ca*. P. pruni’ and 52.63% to ‘*Ca*. P. asteris’ AY-WB. Moreover, homologues having 100% sequence identity and annotated as conserved hypothetical proteins were found in two other draft genomes of ‘*Ca*. P. solani’ strains 284/09 and 231/09 [41]. The other variant showed only 42.27% amino acid sequence identity to the SAP54 of ‘*Ca*. P. asteris’ AY-WB, 41.84% to ‘*Ca*. P. phoenicium’ and 41.67% to a 16SrIII phytoplasma. Hence, we aimed to assess the presence of SAP54-like variant AB2N29_0110 in ‘*Ca*. P. solani’ strains SA-1 and ST19. Amplification by using specific primers followed by sequencing revealed the presence of SAP54-like variant AB2N29_0110 in all three ‘*Ca*. P. solani’ strains (Fig. S1B) with 100% sequence identity. Phylogenetic analyses of nucleotide sequences have shown that this SAP54-like variant AB2N29_0110 belongs to the previously described phyl-C group of phyllogen effectors, while the SAP54-like variant AB2N28_4360 was distantly related to all of the groups described so far (Fig. S2B in Supplementary Material) [42].

Identification of PMUs was based on previously described characteristics of these elements frequently found in phytoplasma genomes [6,7, 9]. These transposon-like features were implicated in genome instability and horizontal gene transfer [10,11]. They often harbour specific genes involved in DNA replication or transposition, such as *tra5, ssb, tmk, hflB*, *dnaB* and *dnaG*. Additionally, genes that encode known effectors and putative secreted proteins are also often found in PMU regions [10,11]. Detailed genome analyses identified the presence of PMU-like regions and elements of various composition and size. In ST19, 14 PMU-like regions were identified with sizes ranging from 2532 to 17 548 bp (Table 2). Notably, PMU-like regions were always found to be located at the end of contigs or encompassing an entire contig. Additionally, 12 genes that encode SAP homologues, putative effectors and putative secreted proteins were identified in these regions (Tables 2 and S2 in Supplementary Material). The five SAP homologues include SAP39-like, SAP53-like, SAP54-like, SAP55-like and SAP64-like. Furthermore, a retron-type reverse transcriptase gene, which often associated with repetitive genomic regions, was also identified (Table 2).

**Table 2.** Identification and features of ‘*Candidatus* Phytoplasma solani’ STOL and ST19 strains’ potential mobile units (PMUs)

	‘*Ca.* P. solani strain’	
	STOL	ST19
**No. of PMUs**	6	14
**Largest PMU size (bp**)	11 914	17 548
**Smallest PMU size (bp**)	4474	2532
**AY-WB SAP-like effectors**	5	5
**Putative effectors**	0	2
**Putative secreted proteins**	3	5
***tra5* gene**	4	3
***hflB* gene**	2	8
***dnaB* and *dnaG* genes**	4	6
**retron-type reverse transcriptase gene**	1	1
**phage-related protein gene**	1	0
***ssb* gene**	1	2
***tmk* gene**	3	2
***himA* gene**	1	2

In STOL, only six PMU-like regions were identified, with sizes ranging from 4474 to 11 914 bp (Table 2). These PMU-like regions were more complete and comparable to the PMUs found in ‘*Ca*. P. solani’ SA-1, as well as those identified in ‘*Ca*. P. asteris’ strains AY-WB and OY-M regarding the gene content and order [7]. When compared to ST19, those PMU-like regions in STOL encompassed fewer putative effector and secreted protein genes, as well as fewer multicopy genes such as *hflB*, *dnaB* and *dnaG*, but the number of SAP-like effector genes was the same. Also, a phage-related protein gene encoding YqaJ-like viral recombinase was identified (Table 2). Phylogenetic analyses of selected PMU-related genes present in multiple copies (*tra5, hflB, dnaB* and *dnaG*) revealed distinct origins of gene copies in all of the four selected genes. Some of the sequences clustered with those from closely related phytoplasmas such as ‘*Ca*. P. australiense’ and ‘*Ca*. P. asteris’, while some of the sequences clustered with phylogenetically distant ‘*Ca*. P. mali’. Furthermore, some of the sequences were found to be unique to ‘*Ca*. P. solani’, particularly *tra5*. Also, multicopy genes from ST19 showed more diversification than those from STOL, particularly *hflB*, *dnaB* and *dnaG* (Fig. S3 in Supplementary Material).

## Discussion

Phytoplasmas encompass a diverse group of pathogens affecting numerous plant species and causing significant damage in agriculture worldwide. While economic importance is increasing, studies related to their pathogenicity mechanisms are still hindered by the inability of axenic cultivation. Nevertheless, the rise of the metagenomic era delivered new tools for genome sequencing of uncultivated microbes. The genomic analysis facilitated the study of potential virulence factors such as effectors that phytoplasmas use to successfully manipulate their plant and insect hosts.

In our study, we were on the quest towards the understanding of a versatile pathogen ‘*Ca*. P. solani’. We conducted genome sequencing of two strains involved in different epidemiological cycles and transmitted by different insect vectors, namely ST19 and STOL, to provide novel genomic information. As in the previous genomic study of ‘*Ca*. P. solani’ SA-1 [7], full assembly of the chromosome was not successful, despite incorporating long reads generated from the ONT platform. This difficulty is likely due to the presence of repetitive sequences and PMUs. Nevertheless, comparison of the gene numbers with other complete assemblies of phytoplasma genomes suggested that the two new drafts from this study have high levels of completeness (Table 1). Our comparative analyses of total gene content identified a substantial number of core genes shared by all representative phytoplasmas, as well as those characteristic for ‘*Ca*. P. solani’ (Fig. 2). Also, our results provide further quantitative estimates of genome size variations among ‘*Ca*. P. solani’ strains. Previously, based on pulsed-field gel electrophoresis experiments, the within-species chromosome size variation was estimated to be ranging from 860 to 1350 kbp [8]. Among the three strains with genome assemblies available, STOL exhibited characteristics of a more reduced genome than ST19. Whole genome alignment showed that STOL has a greater collinearity with SA-1 and has fewer strain-specific regions and repetitive elements such as PMUs (Fig. 1 and Table 2). Accordingly, in the comparative analyses among these three strains, no STOL-specific gene was found. However, 30 gene clusters mainly related to PMU-like regions were shared between STOL and ST19 but absent in SA-1, further corroborating the role of these mobile genetic elements in the diversification among these strains. The genome reduction observed in STOL, together with the presence of fewer PMU-like regions, was previously noticed in some other phytoplasma genomes and correlated with fewer numbers of insect vectors and plant hosts [7,43, 44]. For example, both ‘*Ca*. P. mali’ and FD phytoplasma are transmitted by only one specific insect vector species and specialized for only one plant species (apple and grapevine, respectively). These two phytoplasmas have a genome size of approximately 602 kbp and 654 kbp, respectively. Furthermore, these two phytoplasmas also have fewer annotated hypothetical proteins, lower levels of genome plasticity and no intact PMUs (Table 1) [43,44]. Moreover, the maize bushy stunt phytoplasma (MBSP) belonging to ‘*Ca*. P. asteris’ has a genome size of 576 kbp, which is much smaller compared to other ‘*Ca*. P. asteris’ strains with genome sequences [4] or chromosome size estimates [8]. This MBSP is restricted to maize and transmitted exclusively by insect *Dalbulus maidis*. It was hypothesized that they co-evolved during the process of maize domestication [45]. Similarly, *‘Ca*. P. solani’ STOL is transmitted to maize only by *Reptalus panzeri* and is associated with maize redness disease [46]. However, by the same insect vector, it can be transmitted to other plants, such as grapevine [47]. On the other hand, *‘Ca*. P. solani’ ST19 was transmitted by *Hyalesthes obsoletus*, a primary natural vector of *‘Ca*. P. solani’ and has a broader plant host range, including many crops and natural reservoir species. Therefore, it is involved in much more complex epidemiological networks, particularly in grapevine diseases [24,48]. Thus, we hypothesize that similar to the patterns observed among ‘*Ca*. P. asteris’ strains, specificity regarding insect vectors and plant host, as well as the co-evolution history, could contribute to the diversity of genome sizes among ‘*Ca*. P. solani’ strains.

Since the first studies of phytoplasma effectors [12,13], most works focused on those identified in ‘*Ca*. P. asteris’ strains AY-WB and OY. However, *‘Ca*. P. solani’ effectors are not well studied. Although some putative effectors of *‘Ca*. P. solani’ have been previously identified [7,49], systematic studies of effector diversity among strains are lacking. Hence, in this current study, we aimed to address this issue. In the draft genomes of ‘*Ca*. P. solani’ strains ST19 and STOL, bioinformatic tools enabled prediction of 28 and 26 putative effector and secreted protein genes, which are fewer compared to the 38 identified in strain SA-1 (Table S2) [7]. Detailed comparative analyses have pointed out that some of the predicted SAP-like homologues and other putative effectors were found in all of the analysed ‘*Ca*. P. solani’ strains (Table S2 in Supplementary Material) and shared very high sequence identity with sequences of other ‘*Ca*. P. solani’ strains available in the NCBI database. Thus, these genes could be considered as species-specific. Furthermore, for two SAP homologues well characterized in ‘*Ca*. P. asteris’, namely SAP11-like and SAP54-like, these genes have 100% sequence identity among all ‘*Ca*. P. solani’ strains examined. Interestingly, for the SAP11 effector of ‘*Ca*. P. asteris’, different variants were found in strains with restricted host range and insect vector transmission and showed differential interactions with plant TCP (teosinte branched 1–cycloidea–proliferating cell factor) transcription factors. These findings suggest that SAP11 evolution is linked to the phytoplasma host range [50]. However, this is not the case with the SAP11-like homologue from *‘Ca*. P. solani’, although the two species are closely related (Fig. S2A in Supplementary Material). Moreover, the functional studies of SAP11-like homologue from *‘Ca*. P. solani’ showed that it interacts with plant transcription factors AtTCP2 and AtTCP4 and alters the morphology of transformed *Arabidopsis* plants, indicating that mechanisms of SAP11 and its homologues might be conserved [51]. Among the predicted putative effectors and hypothetical proteins of ‘*Ca*. P. solani’, we also identified many strain-specific ones (Fig. 1b; Table S2 in Supplementary Material) [7], including a new variant of SAP54-like (AB2N28_4360) that is distantly related to all of the described phylogenetic groups of phyllogen effectors (Fig. S2B in Supplementary Material) [42]. However, due to the limited availability of ‘*Ca*. P. solani’ genome assemblies and their incompleteness, the extent and the roles of strain-specific effectors need to be evaluated in the future.

Previous works inferred that PMU-like regions contribute to phytoplasma genome plasticity and horizontal gene transfer; therefore, they may promote diversification and adaptation [6,7, 9, 10]. In both of the newly sequenced genomes within this study, PMU-like regions were characterized and found to harbour genes that encode putative effectors and secreted proteins (Table 2). Notably, the number of PMU-like regions correlates with the number of predicted effector and secreted protein genes (Table 2). Moreover, PMU-like regions identified in strain STOL were similar in gene content and order to those characterized in ‘*Ca*. P. solani’ SA-1 and ‘*Ca*. P. asteris’ strains [7], which further suggested the correlation of the presence of repetitive elements with adaptation and specialization to specific insect vectors. Also, molecular phylogeny of selected PMU-related genes demonstrated more variability in ST19 than in STOL, further emphasizing the role of PMUs in the diversification of ‘*Ca*. P. solani’ strains (Fig. S3 in Supplementary Material).

In conclusion, our results provide novel datasets on the diversification of ‘*Ca*. P. solani’ genomes, including the reference strain STOL. These datasets set a base for future functional studies of putative effectors and their interactions with host targets, which could facilitate deciphering the pathogenicity strategies of this successful and versatile pathogen. Furthermore, the evolution of specificity to insect vector species and plant host, as well as the contribution of this factor to the diversity of genome size among ‘*Ca*. P. solani’ strains, remains to be further elucidated in the future.

## Supplementary material

10.1099/mgen.0.001401 Supplementary Material 1.

## Abbreviations

AF: alignment fraction
ANI: average nucleotide identity
CDS: coding sequence
MBSP: maize bushy stunt phytoplasma
OGRI: overall genome relatedness index
ONT: Oxford Nanopore Technologies
PMU: potential mobile unit
SAP: secreted AY-WB proteins
