# Supplementary material for: Sequencing and comparative analyses of ‘Candidatus Phytoplasma solani’ genomes reveal diversity of effectors and potential mobile units
Source: Microb Genom. 2025 Apr 28;11(4):001401. doi: 10.1099/mgen.0.001401 (PMC12047186; doi:10.1099/mgen.0.001401)
Supplement: Supplementary Material 1. [file mgen-11-01401-s001.pdf]

Supplementary Table 1. List of newly designed primers and PCR conditions used for amplification of *SAP11-like* and *SAP54-like* genes from *'Ca.* P. solani' strains.

**Primer**	**PCR conditions**
SAP11_2F<br>5' - TCAAAGGAGTATTTATTAAATGTTT- 3'	Initial denaturation at 94 °C, 4 min; 35 cycles of denaturation at 94°C, 1 min, annealing at 50 °C, 1 min, and extension 68 °C, 1 min; and final extension at 68 °C, 7 min
SAP11_336R<br>5' - GCTGTCTAGAAGAAGAACCTT- 3'	
SAP54_40F<br>5' – TGTTTGTTTATTTTCTTGGGA – 3'	
SAP54_319R<br>5' – TTTTCAGTTGTTCTTTTTGA – 3'	

Supplementary Table 2. Lists of putative secreted proteins and effectors identified in *'Ca*. P. solani' strains (A) STOL and (B) ST19.

A)

Locus_tag	Description
AB2N29_0110	AYWB SAP54-like protein
AB2N29_0150	AYWB SAP44-like protein
AB2N29_0230	putative effector
AB2N29_0300	putative secreted protein
AB2N29_0750	putative secreted protein
AB2N29_1220	putative secreted protein
AB2N29_1510	putative secreted protein
AB2N29_1970	putative secreted protein
AB2N29_2630	putative secreted protein
AB2N29_2660**	AYWB SAP09, SAP39-like protein
AB2N29_2690	putative secreted protein
AB2N29_2710	putative secreted protein
AB2N29_3490	putative secreted protein
AB2N29_3920**	AYWB SAP50-like protein
AB2N29_3960**	putative effector
AB2N29_4030	ABC-type sugar transport system, substrate-binding protein
AB2N29_4130**	AYWB SAP53-like protein

AB2N29_4140	AYWB SAP19, SAP40-like protein
AB2N29_4160	putative secreted protein
AB2N29_4480	AYWB SAP59-like protein
AB2N29_4630	AYWB SAP65-like protein
AB2N29_4650	hypothetical protein
AB2N29_4780	putative secreted protein
AB2N29_5120**	AYWB SAP61-like protein
AB2N29_5460	putative secreted protein
AB2N29_5480	putative secreted protein

B)

**Locus_tag**	**Description**
AB2N28_0500**	AYWB SAP09, SAP39-like protein
AB2N28_0630	putative secreted protein
AB2N28_1340	putative secreted protein
AB2N28_1570	AYWB SAP59-like protein
AB2N28_1710	ABC-type sugar transport system, substrate-binding protein
AB2N28_2310	putative secreted protein
AB2N28_2350**	AYWB SAP53-like protein
AB2N28_2360	AYWB SAP55-like protein
AB2N28_2650	putative secreted protein
AB2N28_3520	AYWB SAP25, SAP64-like protein
AB2N28_3620	putative secreted protein

AB2N28_3630	ATP-dependent Zn protease
AB2N28_3750	ATP-dependent Zn protease
AB2N28_4080*	putative effector
AB2N28_4360	AYWB SAP54-like protein
AB2N28_4840	putative secreted protein
AB2N28_4870	putative secreted protein
AB2N28_4880	putative secreted protein
AB2N28_5590	putative secreted protein
AB2N28_5600**	AYWB SAP50-like protein
AB2N28_5640**	putative effector
AB2N28_5650	putative secreted protein
AB2N28_5710*	putative effector
AB2N28_5730	hypothetical protein
AB2N28_5860	putative secreted protein
AB2N28_5950	variable membrane protein
AB2N28_6190*	putative effector
AB2N28_6480**	AYWB SAP61-like protein

*putative strain-specific effectors

**putative species-specific effectors

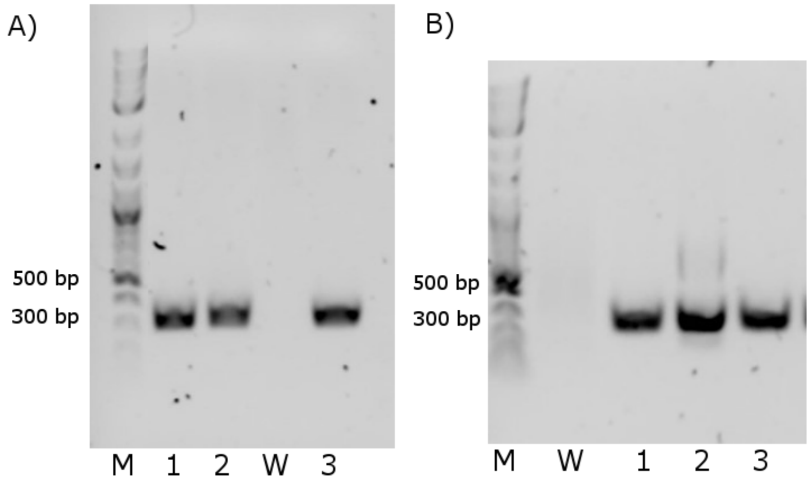

Supplementary Figure 1. Confirmation of (A) *SAP11-like* and (B) *SAP54-like* genes from '*Ca.* P. solani' strains by PCR and gel electrophoresis. Amplicons of 336 bp and 354 bp were obtained for these two genes. Labels for gel lanes: M, GeneRuler 1 kb Plus DNA Ladder (ThermoFisher Scientific); W, negative control, 1, Strain ST19; 2, Strain STOL; 3, Strain SA-1. For the DNA Ladder, a thick band corresponding to 500 bp and a thin band corresponding to 300 bp are labelled.

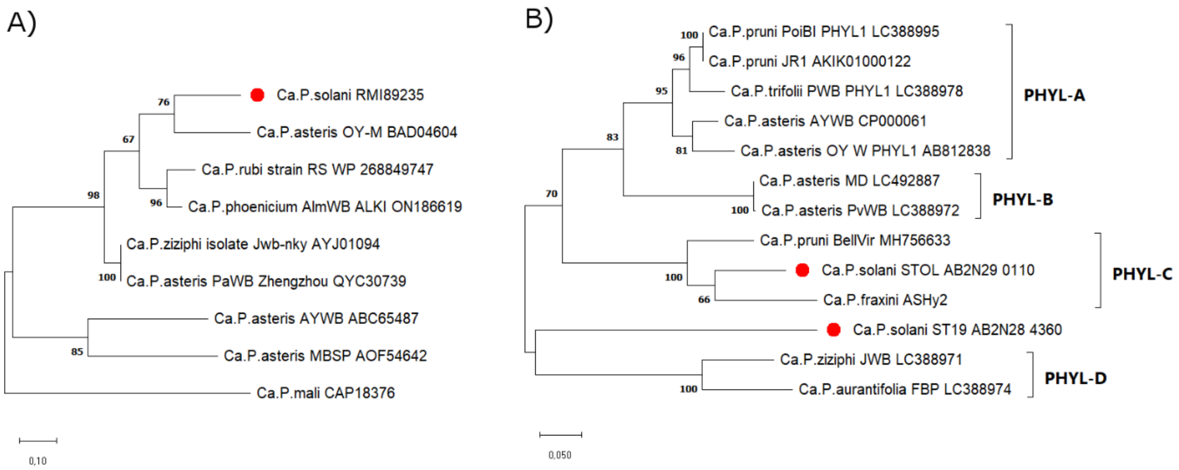

Supplementary Figure 2. Evolutionary relationships of (A) SAP11-like and (B) SAP54-like effectors among phytoplasmas. The phylogenetic trees were inferred by maximum likelihood method based on (A) amino-acid or (B) nucleotide datasets. Bootstrap values based on 500 replicates are indicated with numbers next to the internal branches. The scale bar indicates the number of substitutions per site. GenBank accession numbers are provided next to the name of each species or strain. Sequences of '*Ca*. P. solani' strains STOL and ST19 are marked with red dots.

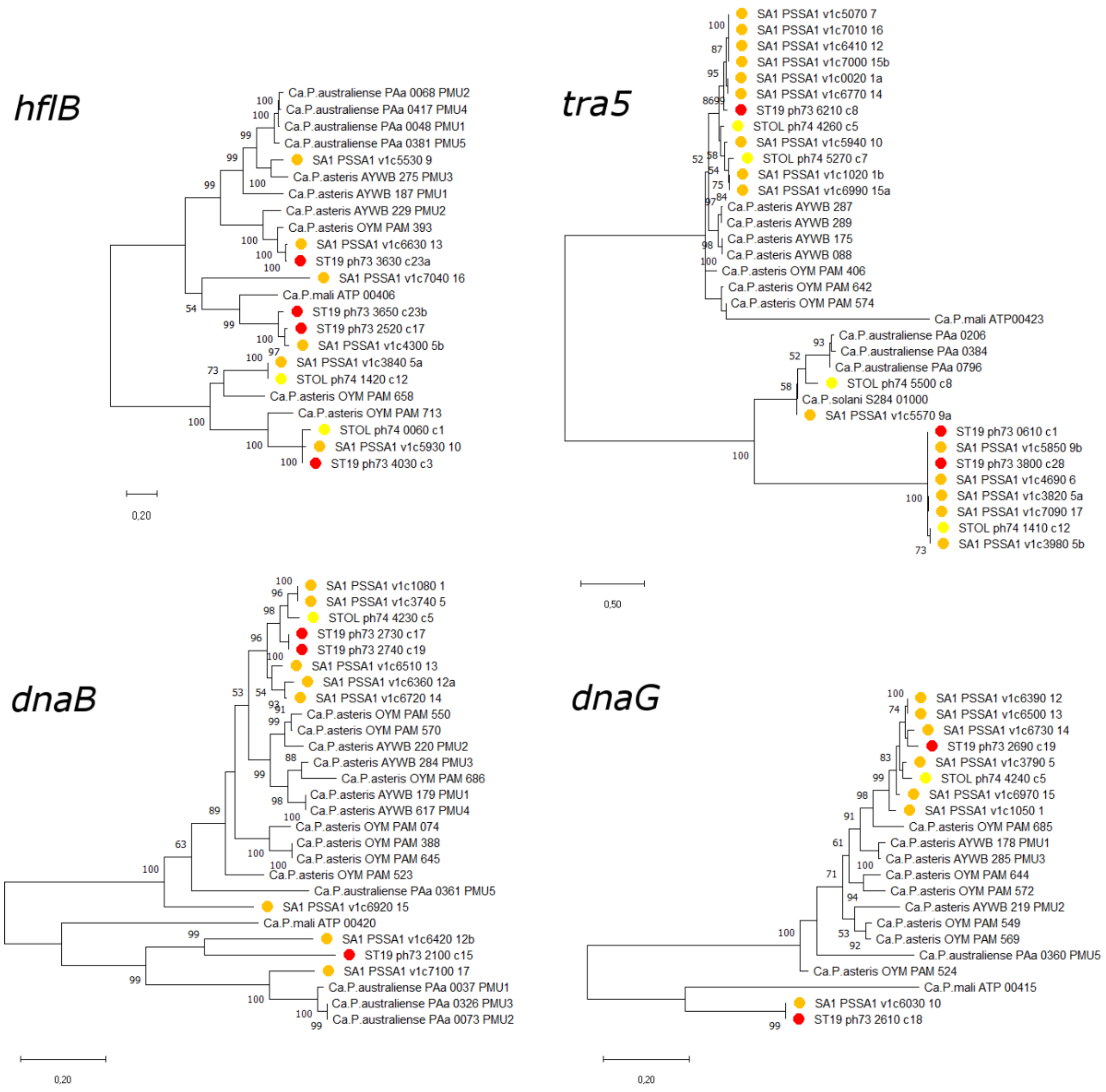

Supplementary Figure 3. Molecular phylogeny of phytoplasma PMU-associated genes *hflB, tra5, dnaG*, and *dnaB.* Phylogenetic trees were inferred by maximum likelihood method. Bootstrap values based on 500 replicates are indicated with numbers next to the internal branches. The scale bar indicates the number of substitutions per site. GenBank accession numbers are provided in Table 1 and the locus tags are labelled next to the name of each species or strain. The sequences derived from '*Ca.* P. solani' strains are indicated with colour dots (red, ST19; orange, SA-1; yellow, STOL).
